# Altered reward processing underpins emotional apathy in dementia

**DOI:** 10.3758/s13415-022-01048-2

**Published:** 2022-11-23

**Authors:** Stephanie Wong, Grace Wei, Masud Husain, John R. Hodges, Olivier Piguet, Muireann Irish, Fiona Kumfor

**Affiliations:** 1 College of Education, Psychology & Social Work, Flinders University, Adelaide, SA 5042 Australia; 2 Brain & Mind Centre, The University of Sydney, Sydney, NSW 2050 Australia; 3 School of Psychology, The University of Sydney, Sydney, NSW 2050 Australia; 4 Department of Experimental Psychology, University of Oxford, Oxford, OX1 2JD UK; 5 Sydney Medical School, The University of Sydney, Sydney, NSW 2050 Australia

**Keywords:** Apathy, Emotional apathy, Reward, Motivation, Dementia

## Abstract

**Introduction:**

While apathy is broadly defined as a loss of motivation, it is increasingly recognised as a multidimensional syndrome spanning executive, emotional, and initiation domains. Emotional apathy is purportedly driven by deficits in using socioemotional rewards to guide behaviour, yet the link between these symptoms and reward processing, and their common neural correlates, has not been directly examined.

**Methods:**

Sixty-four patients (33 behavioural-variant frontotemporal dementia, 14 Alzheimer’s disease, 8 semantic dementia, 6 progressive nonfluent aphasia, 3 logopenic progressive aphasia) were classified into high (HEA; n = 36) and low (LEA; n = 28) emotional apathy groups based on emotional apathy subscale scores on the Dimensional Apathy Scale. Patients and age-matched healthy controls (n = 27) performed an instrumental reward learning task where they learned to associate cues with either social or monetary outcomes.

**Results:**

HEA patients showed impaired learning on both the social and monetary reward conditions, relative to LEA patients (*p* = 0.016) and controls (*p* = 0.005). Conversely, the LEA group did not differ from controls (*p* = 0.925). Importantly, multiple regression analyses indicated that social reward learning significantly predicted emotional apathy. Voxel-based morphometry analyses revealed that emotional apathy and social reward learning were both associated with orbitofrontal cortex, ventral striatum, and insula atrophy.

**Discussion:**

Our results demonstrate a unique link between impaired social reward learning and emotional apathy in dementia and reveal a shared neurobiological basis. Greater understanding of these neurocognitive mechanisms of reward processing will help improve the identification of emotional apathy in dementia and inform the development of novel interventions to address these symptoms.

**Supplementary Information:**

The online version contains supplementary material available at 10.3758/s13415-022-01048-2.

## Introduction

Apathy is broadly defined as a loss of motivation and goal-directed behaviour (Marin, 1991), which severely limits the ability to live independently, and to perform everyday activities, such as planning, maintaining social relationships, and self-care. Apathy is common in dementia, affecting up to ninety percent of patients over the disease course (Chow et al., 2009; Steinberg et al., 2008; van Reekum et al., 2005). It profoundly impacts dementia patients and their families and is associated with greater functional deterioration, increased carer burden, and earlier mortality (Hongisto et al., 2018; Lansdall et al., 2019; Massimo et al., 2014; Wong et al., 2020).

While many previous studies have considered apathy as a unitary construct, the multidimensional nature of the apathy syndrome is increasingly recognised. Executive, emotional, and initiation domains of apathy have been hypothesised, each proposed to be supported by distinct cognitive and neural mechanisms (Dickson and Husain, 2022; Johnson and Kumfor, 2018; Levy and Dubois, 2006; Marin, 1991; Radakovic and Abrahams, 2018). We focus on emotional apathy, which is characterised by emotional blunting, reduced empathy, and altered social interactions (Johnson and Kumfor, 2018). Thus far, emotional apathy has been examined using self- or informant-report scales with questions, such as “they struggle to empathise with other people” or “they are unconcerned about how others feel about their behaviour” (Ang et al., 2017; Radakovic et al., 2016). Using the Dimensional Apathy Scale (Radakovic et al., 2016) and similar measures, we and others have shown that emotional apathy is present in varying degrees across dementia subtypes, including behavioural-variant frontotemporal dementia (Wei et al., 2019), Alzheimer’s disease (Radakovic et al., 2017; Wei et al., 2019), semantic dementia, progressive nonfluent aphasia, and logopenic progressive aphasia (Quang et al., 2021). Despite the prevalence of emotional apathy across different dementia syndromes, knowledge about the underlying mechanisms of these symptoms is scant. A transdiagnostic approach, which examines the cognitive and neural bases of these symptoms across patients with different dementia diagnoses, may provide important insights into shared mechanisms. Given the pervasiveness of apathy symptoms in dementia, such knowledge may provide a new evidence base for the development of tailored, patient-centered therapeutic interventions targeting emotional apathy (Husain and Roiser, 2018).

It has been proposed that emotional apathy stems from an inability to use social and emotional cues or outcomes to modify behaviour (Levy and Dubois, 2006). Specifically, it has been theorised that impaired learning from social and emotional rewards leads to reduced emotional reactivity and social engagement (Levy and Dubois, 2006; Viskontas et al., 2007; Wong et al., 2018). To date, however, these claims have not been empirically tested. It is also unclear whether breakdown in the *perception* of socioemotional reward or deficits in the use of such rewards to modify behaviour (i.e., reward *learning*) contributes to emotional apathy in dementia. With regards to the former, deficits in emotion perception are well-documented in behavioural-variant frontotemporal dementia (Kumfor and Piguet, 2012), semantic dementia, and progressive nonfluent aphasia (Couto et al., 2013; Kumfor et al., 2011) but are less common in the early stages of Alzheimer’s disease (Bertoux et al., 2015). The link between emotion perception and emotional apathy in these patient groups, however, has not been examined. Furthermore, while existing studies on reward processing have mainly used nonsocial (e.g., monetary) rewards, one study (Perry et al., 2015) contrasted sensitivity to social and monetary rewards and losses in behavioural-variant frontotemporal dementia and Alzheimer’s disease patients. Behavioural-variant frontotemporal dementia patients were found to react faster to gain monetary reward than to avoid monetary loss, whereas Alzheimer’s disease patients reacted faster to gain social reward than to avoid negative social feedback (Perry et al., 2015). This raises a fundamental question: is emotional apathy underpinned by a *general* impairment in reward learning or is emotional apathy related to a specific impairment in *social* reward learning? To our knowledge, the relationship between social reward learning and emotional apathy has not been investigated.

Converging evidence from neuroimaging studies of dementia patients has shown that atrophy in orbito-medial prefrontal regions is associated with symptoms of emotional apathy (Kumfor et al., 2018; Quang et al., 2021; Wei et al., 2019). This region has also been implicated in modifying behaviour as a function of reward. For example, patients with orbito-medial prefrontal lesions are impaired on instrumental reward learning tasks and decision-making tasks where participants are required to learn to make optimal choices or decisions based on reward-related feedback (e.g., winning or losing money) over multiple learning trials (Bechara, 2004; Bechara et al., 2000; Hornak et al., 2004). Similarly, previous studies in Parkinson’s disease (O'Callaghan et al., 2013), frontotemporal dementia (including a mixed sample of behavioural-variant frontotemporal dementia, semantic dementia, and progressive nonfluent aphasia) (Dalton et al., 2013), behavioural-variant frontotemporal dementia, and Alzheimer’s disease (Kloeters et al., 2013) have demonstrated links between impaired reward learning and decision-making, and atrophy in the orbito-medial prefrontal cortices and striatum. Given that these studies have mostly used monetary rewards, the structural neural correlates of social versus monetary reward learning in patients with dementia, and their overlap with neural correlates of emotional apathy remain unclear.

The objective of this study was to investigate the mechanisms underpinning emotional apathy in dementia by examining performance on emotion perception and social and monetary reward learning tasks, with concurrent structural neuroimaging analyses. In recognition of the pervasiveness of apathy symptoms across dementia syndromes, we took a transdiagnostic approach including behavioural-variant frontotemporal dementia, Alzheimer’s disease, semantic dementia, progressive nonfluent aphasia, and logopenic progressive aphasia patients who were classified into high or low emotional apathy subgroups based on scores from the Dimensional Apathy Scale (Radakovic et al., 2016). We hypothesised that patients with high emotional apathy would show greater impairments in reward learning, particularly for social rewards, as well as poorer emotion perception. We also expected that emotion perception and social reward learning performance would predict the severity of emotional apathy to varying degrees. Finally, we hypothesised that, irrespective of clinical diagnosis, both emotional apathy and reward learning performance would scale with the magnitude of orbito-medial prefrontal cortex and striatal atrophy, with shared neural correlates in these regions underpinning both emotional apathy and social reward learning.

## Materials and methods

### Participants

Sixty-four dementia patients (33 behavioural-variant frontotemporal dementia, 14 Alzheimer’s disease, 8 semantic dementia, 6 progressive nonfluent aphasia, and 3 logopenic progressive aphasia) and 27 healthy controls were recruited. All dementia patients fulfilled relevant clinical diagnostic criteria (Gorno-Tempini et al., 2011; McKhann et al., 2011; Rascovsky et al., 2011). Disease duration was calculated as the number of years elapsed since the reported onset of symptoms. The Frontotemporal Dementia Rating Scale (Mioshi et al., 2010) was used to estimate clinical disease severity. All participants underwent general cognitive screening using the Addenbrooke’s Cognitive Examination-III (ACE-III) (Hsieh et al., 2013). Age-matched healthy controls were recruited from the Frontier volunteer registry and scored >88 on the ACE-III (Hsieh et al., 2013). The depression subscale of the Depression Anxiety and Stress Scale (Lovibond and Lovibond, 1995) was used to assess self-reported symptoms of depression. Exclusion criteria for all participants included current or prior history of mental illness, significant head injury, movement disorders, cerebrovascular disease (stroke, transient ischaemic attacks), alcohol or other drug abuse, and limited English proficiency.

### Emotional apathy assessment

The Dimensional Apathy Scale (DAS) is a 24-item questionnaire that was developed to assess executive, emotional, and initiation apathy (Radakovic and Abrahams, 2014). The DAS was completed by carers/informants for patients and self-rated in controls. Each item was rated using a four-point Likert scale (0 = hardly ever, 1 = occasionally, 2 = often, and 3 = almost always/always), with higher scores indicating more severe emotional apathy (maximum score = 24). Patients were classified as either showing high emotional apathy (HEA) or low emotional apathy (LEA) using the previously published cutoff (score ≥15) for clinically significant emotional apathy (Radakovic et al., 2016).

### Emotion perception assessment

The Facial Affect Selection Test (Kumfor et al., 2014; Miller et al., 2012) was used as a measure of facial emotion perception. Participants were shown an array of seven faces of the same person. Each face showed a different emotional expression (happy, angry, sad, surprise, fear, disgust, or neutral). Participants were asked to point to the verbally cued emotional expression (e.g., “point to the happy face”). One point was given for each correct answer (maximum score = 42).

### Social and monetary reward learning task

**Fig. 1:**
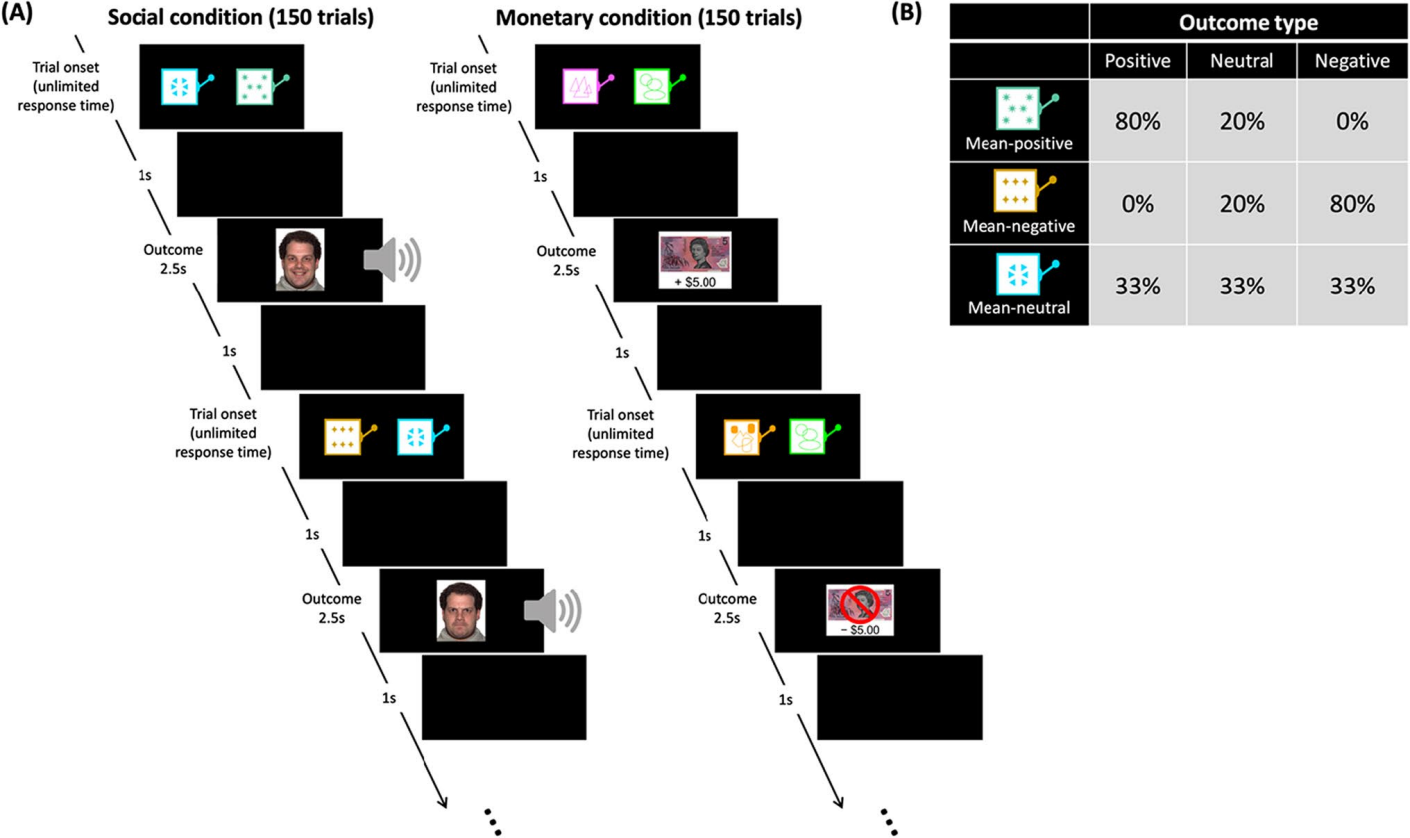
Social and monetary reward learning task. (**A**) Timeline of the social and monetary reward learning trials. On each trial, participants made a binary choice between two slot machines: the mean-neutral slot machine versus either the mean-positive or the mean-negative slot machine (left-right placement randomised). Following selection of either the left or right slot machine (by pressing the left or right arrow key on the keyboard), the trial outcome was displayed for 2.5 seconds before the screen progressed to the next trial. Trials across the social and monetary conditions were identical except for the nature of the outcomes: trials in the social condition revealed happy, neutral, or angry faces accompanied with spoken words of the same valence and prosody (e.g., excellent, table, or stupid), whereas trials in the monetary condition had a gain/loss of +$5, $0, or −$5. **(B)** Probabilistic outcome distributions for the mean-positive, mean-negative and mean-neutral slot machines. The same distributions of outcome types (positive, negative, neutral) were used in the social and monetary conditions. The actual appearance of the slot machines was distinct between social and monetary conditions and counterbalanced across participants. Participants were not informed of the outcome type probabilities of each slot machine. They were instructed to learn by trial and error which was the “best outcome.” Participants were led to believe that they would be paid a proportion of their earnings from the monetary condition but received the same final amount for participation ($20)

Participants completed two structurally identical conditions of a computerised instrumental reward learning task, adapted from Lin, Rangel, et al. (2012b), one with social outcomes and another with monetary outcomes. See Figure 1 caption for a full description of the task. In each condition, participants made 150 binary choices between two slot machines, which were probabilistically associated with mean-positive, mean-negative, or mean-neutral outcomes. Of the 150 trials, 75 trials involved choosing between the mean-positive and mean-neutral slot machine (i.e., positive trial), and 75 trials involved choosing between the mean-neutral and mean-negative slot machine (i.e., negative trial). Positive and negative trials were randomised.

The experimental task was modified for older adults and patients with dementia by increasing the number of choice trials per condition and removing the 2.5-s response time limit. For each trial, participants’ responses were converted to a binary choice score where 1 = optimal choice and 0 = nonoptimal choice. Following the learning phase, participants completed a choice test, which included eight further trials of the social and monetary conditions under extinction (i.e., without presentation of outcomes). The order of conditions was counterbalanced across participants. Following the choice test, participants were asked to rate the pleasantness of the social and monetary stimuli used in the learning task. Ratings were provided on a 7-point Likert scale ranging from “extremely unpleasant” to “extremely pleasant.”

### Statistical analyses

Data were analysed by using SPSS v. 24.0 (SPSS Inc., Chicago, IL). Normally distributed variables, as determined by Shapiro-Wilks tests, were compared across groups by using ANOVAs followed by Sidak *post hoc* tests, or independent samples *t*-tests. Data that were not normally distributed were analysed using Kruskal-Wallis tests followed by *post hoc* pairwise comparisons, using Dunn’s (1964) procedure with a Bonferroni correction for multiple comparisons. Chi-squared tests were used to compare sex distribution and emotional apathy subgroup (LEA, HEA) distribution across groups.

**Fig. 2:**
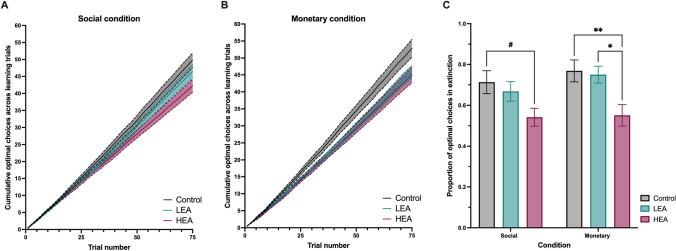
Social and monetary reward learning task performance. **(A)** Plot of cumulative optimal choices across learning trials (i.e., learning rate) in the social condition for Controls, low emotional apathy (LEA), and high emotional apathy (HEA) patients. **(B)** Plot of cumulative optimal choices across learning trials (i.e., learning rate) in the monetary condition for Controls, LEA, and HEA patients. Steeper slopes indicate faster learning. **(C)** Overall social and monetary reward learning performance, denoted by proportion of optimal choices made in extinction on the post-learning choice test for the social and monetary conditions across groups. See also Supplementary Figure 1 for individual post-learning choice test performance according to diagnosis group. Error bars represent standard error of the mean. **p < 0.01; *p < 0.05; ^#^trend towards significant effect

To investigate group differences in learning rate, the cumulative number of optimal choices trial by trial for each condition (averaged across positive and negative trials; Figure 2a) was plotted for each participant and then averaged for each group. Best-fit lines were calculated for each group by using simple linear regression. The slopes of the best-fit lines were contrasted between groups (controls, LEA, HEA) using separate ANOVAs for the social and monetary conditions, followed by Sidak *post hoc* tests. Next, reward learning performance was operationalised in terms of the proportion of optimal choices made during the choice test (i.e., without further outcome delivery), ensuring that participants’ choices reflected their knowledge of the stimulus-outcome contingencies acquired during the learning phase. The effects of group and condition on choice test performance were investigated using repeated measures ANOVA with condition (social, monetary) as the within subjects measure and group (controls, LEA, HEA) as the between subjects measure. As stimuli pleasantness ratings were not normally distributed, group comparisons were conducted using nonparametric equivalents.

Finally, to empirically test the notion that deficits in using socio-emotional outcomes to guide behaviour give rise to emotional apathy (Levy and Dubois, 2006) and to explore the potential influence of emotion perception deficits on emotional apathy, we conducted stepwise linear regression analysis (backward method) to determine the extent that emotion perception performance and social and monetary reward learning performance on the choice test predicted emotional apathy, taking into account patient diagnosis, disease duration, and general cognition (ACE-III). Additional stepwise linear regression analyses were conducted by using the same predictor variables to investigate their relationships with cognitive apathy and initiation apathy scores from the DAS. Statistical significance for all analyses was set at *p* < 0.05.

### Neuroimaging acquisition and analyses

Eighty-five, whole-brain structural MRI scans (26 control, 30 behavioural-variant frontotemporal dementia, 14 Alzheimer’s disease, 6 semantic dementia, 6 progressive nonfluent aphasia, 3 logopenic progressive aphasia) were available for analysis. Scans were unavailable for three participants (1 control, 1 behavioural-variant frontotemporal dementia, 1 semantic dementia), whereas three further participants (2 behavioural-variant frontotemporal dementia, 1 semantic dementia) were excluded due to excessive movement artifacts. Scans were collected by using a 3 T GE scanner with the following protocol: coronal orientation, matrix 256 × 256, 200 slices, 1-mm^2^ in-plane resolution, 1-mm slice thickness, echo time/repetition time: 2.5/6.7 ms, flip angle 8°.

The MRI data were analysed using FSL-VBM, a VBM analysis (Ashburner and Friston, 2000; Good et al., 2001), which is part of the FMRI software package (Smith et al., 2004). Following brain extraction, tissue segmentation was performed by using FMRIB’s Automatic Segmentation Tool (Zhang et al., 2001). The resulting grey matter partial volume maps were aligned to the Montreal Neurological Institute standard space (MNI52) by using the nonlinear registration approach with FNIRT (Anderson et al., 2007a, 2007b), which uses a b-spline representation of the registration warp field (Rueckert et al., 1999). A study-specific template was created to which the native grey matter images were nonlinearly re-registered. Modulation of the registered partial volume maps was performed by dividing them by the Jacobian of the warp field. The modulated, segmented images were smoothed with an isotropic Gaussian kernel with a sigma of 3 mm.

Voxel-wise general linear models (GLM) were used to investigate differences in grey matter intensity via permutation-based nonparametric testing (Nichols and Holmes, 2002) with 5,000 permutations per contrast. As a first step, group differences in grey matter intensity were tested for significance at *p* < 0.005, corrected for multiple comparisons via Family-Wise Error correction across space. A cluster extent threshold of 200 contiguous voxels was applied for group comparisons.

Next, whole-brain covariate analyses were conducted to explore the relationship between grey matter intensity and emotional apathy and reward learning in the patient cohort only (n = 59). A General Linear Model was constructed, with DAS emotional apathy, social reward choice test, and monetary reward choice test scores entered simultaneously into the design matrix to determine common regions associated with 1) emotional apathy *and* social reward learning [−1,1]; 2) emotional apathy *and* monetary reward learning [−1,1]; 3) social reward learning *and* monetary reward learning [1,1]; and 4) emotional apathy, social reward learning, and monetary reward learning [−1,1,1]. Separate General Linear models were also conducted with emotional apathy, social reward learning, and monetary reward learning scores entered individually into the design matrix to determine regions of brain atrophy associated with each variable alone (see Supplementary Materials). To boost power to detect meaningful signal, while controlling for false positives, clusters were extracted voxel-wise and corrected using a false discovery rate of q = 0.05 (Bennett et al., 2009). This yielded corrected *p*-values < 0.005 from the data. In addition, a conservative cluster extent threshold of 100 contiguous voxels was applied to reduce the likelihood of false positives (Lieberman and Cunningham, 2009). Anatomical locations of significant results were overlaid on the MNI standard brain, with maximum coordinates provided in MNI stereotaxic space. Anatomical labels were determined with reference to the Harvard Oxford probabilistic cortical and subcortical atlases.

## Results

Demographic and background clinical variables for each diagnosis group are reported in Supplementary Table 1. Based on the DAS emotional apathy cut-off score (Radakovic et al., 2016), 36 patients were classified as “low emotional apathy” (LEA; 12 behavioural-variant frontotemporal dementia, 12 Alzheimer’s disease, 5 semantic dementia, 5 progressive nonfluent aphasia, and 2 logopenic progressive aphasia), and 28 patients were classified as “high emotional apathy” (HEA; 21 behavioural-variant frontotemporal dementia, 2 Alzheimer’s disease, 3 semantic dementia, 1 progressive nonfluent aphasia, and 1 logopenic progressive aphasia). A significant difference in the distribution of patient diagnosis across groups was observed (χ^2^ = 12.289, *p* = 0.015) with a higher proportion of behavioural-variant frontotemporal dementia than Alzheimer’s disease patients in the HEA group (*p* = 0.002). All other *post hoc* comparisons of patient diagnosis distributions were not statistically significant. LEA, HEA, and controls did not differ in age (*p* = 0.283) or years of education (*p* = 0.118) (Table 1). A significant difference in sex distribution was observed (χ^2^ = 11.707, *p* = 0.003), with more males in the HEA group than in the control group (*p* < 0.001). The LEA and HEA groups did not differ in disease duration (*p* = 0.668), but the HEA group showed greater disease severity on the Frontotemporal Dementia Rating Scale (*t*(57) = 4.738, *p* < 0.001). On the Depression, Anxiety and Stress Scale, self-reported symptoms of depression were significantly higher in both patient groups than in controls (*p* < 0.033), but no significant differences were detected between the LEA and HEA groups (*p* > 0.114).

**Table 1:** Demographic and clinical characteristics for the control and low emotional apathy (LEA) and high emotional apathy (HEA) patient subgroups^1^

	Control	LEA	HEA	Group difference (*p* value)	*Post hoc* group comparisons
N	27	36	28		
Sex (male:female)	10:17	22:14	23:5	0.003	
Age (yr)	65.99 ± 7.62	64.90 ± 8.02	63.04 ± 8.04	0.283	
Education (yr)	13.40 ± 2.72	12.60 ± 3.12	11.93 ± 3.73	0.118	
Clinical diagnosis (bvFTD:AD:SD:PNFA:LPA)^2^	.	12:12:5:5:2	21:2:3:1:1	0.003	bvFTD > AD in the HEA group
Disease duration (yr)	.	5.12 ± 2.57	6.04 ± 5.19	0.668	
FRS Rasch score^3^	.	1.38 ± 1.46	-0.45 ± 1.51	<0.001	LEA > HEA
DASS depression [42]^4^	1.04 ± 1.29	6.59 ± 7.21	11.38 ± 8.94	<0.001	Con < LEA, HEA
ACE-III total [100]^5^	94.30 ± 3.26	78.25 ± 12.31	76.43 ± 12.11	<0.001	Con > LEA, HEA
Emotion perception [42]	39.00 (3.06)	34.77 (4.93)	31.00 (5.08)	<0.001	Con > LEA > HEA
DAS Emotional apathy [24]^6^	7.16 ± 4.01	9.94 ± 3.37	18.96 ± 3.01	<0.001	Con, LEA < HEA
DAS Executive apathy [24]^6^	3.68 (3.80)	11.64 (5.14)	16.89 (5.34)	<0.001	Con < LEA < HEA
DAS Initiation apathy [24]^6^	7.20 (5.13)	13.58 (4.83)	18.00 (3.93)	<0.001	Con < LEA < HEA

^1^Values are mean ± standard deviation. Maximum scores on each measure shown in square brackets.

^2^bvFTD = behavioural-variant frontotemporal dementia; AD = Alzheimer’s disease; SD = semantic dementia; PNFA= progressive nonfluent aphasia; LPA = logopenic progressive aphasia.

^3^FRS = Frontotemporal dementia Rating Scale.

^4^DASS = Depression, Anxiety and Stress Scale; DAS = Dimensional Apathy Scale.

^5^ACE-III = Addenbrooke’s Cognitive Examination, Third Edition.

^6^DAS = Dimensional Apathy Scale.

Missing data: DAS (3 controls), Emotion perception (2 Controls, 6 LEA, 2 HEA).

Both patient groups scored significantly lower than controls on the ACE-III (*p* < 0.001) but did not differ from each other (*p* = 0.767). On the emotion perception test (Facial Affect Selection Test), both patient groups scored significantly lower than controls (LEA *p* = 0.004; HEA *p* < 0.001), with lower performance in the HEA than the LEA group (*p* = 0.04). As expected, the HEA group showed elevated emotional apathy scores relative to the control and LEA groups (*p* < 0.001), with no significant difference between the control and LEA groups (*p* = 0.18). Executive and initiation apathy scores were significantly elevated in both patient groups relative to controls (*p* < 0.001), with higher scores in the HEA compared to LEA group (executive apathy *p* = 0.013; initiation apathy *p* = 0.001).

### Social and monetary reward learning task

#### Learning rate

Figures 2A and B show the averaged cumulative number of optimal choices for each group across the social and monetary learning trials. In the social condition, a significant group difference was observed (*F*(2,6) = 1228.8, *p* < 0.001), with flatter learning slopes in both LEA (*p* < 0.001) and HEA (*p* < 0.001) patients than in controls. Importantly, HEA patients also showed significantly slower learning than LEA patients (*p* < 0.001). In the monetary condition, a significant group difference was also present (*F*(2,6) = 1213.82, *p* < 0.001), with flatter learning slopes in both LEA (*p* < 0.001) and HEA (*p* < 0.001) patients than in controls, and slower learning in HEA than LEA patients (*p* = 0.04).

#### Choice test

The post-learning choice test was performed in extinction (i.e., without further outcome delivery), which served as an indicator of the participants’ knowledge of the stimulus-outcome contingencies acquired during the learning phase (Figure 2C). Analysis of the post-learning choice test scores revealed a main effect of group (*F*(2,83) = 6.264, *p* = 0.003), with HEA patients showing significantly lower accuracy than LEA patients (*p* = 0.016) and controls (*p* = 0.005) averaged across conditions. No difference was observed between LEA patients and controls (*p* = 0.925). The condition effect (*F*(1,83) = 2.355, *p* = 0.129) and group × condition interaction (*F*(2,83) = 0.451, *p* = 0.639) were not significant. *Post hoc* simple effects tests revealed that on the social condition, the HEA patients tended to show lower choice test accuracy than controls (*p* = 0.058), with no difference between LEA and controls (*p* = 0.888) or LEA and HEA (*p* = 0.200). On the monetary condition, the HEA group performed worse than LEA patients (*p* = 0.014) and controls (*p* = 0.009), with no significant difference between the LEA and control group (*p* = 0.990). We re-ran the repeated measures ANOVA with ACE-III and disease duration as covariates to control for potential differences in overall cognition and disease duration, with the results remaining the same. To summarise, the HEA group had worse accuracy in selecting the optimal choice during the choice test, demonstrating poorer learning from rewards across both conditions, whereas the LEA group performed similarly to controls for both monetary and social reward.

#### Pleasantness ratings

The average pleasantness ratings for each group are shown in Table 2. A significant group difference in pleasantness ratings was observed, with HEA patients rating social negative outcomes as more “pleasant” than LEA patients (*p* = 0.025) and controls (*p* = 0.008). No difference in pleasantness ratings were seen for the monetary condition (*p* > 0.483). Furthermore, no significant differences in pleasantness ratings were observed between LEA patients and controls.

**Table 2:** Mean pleasantness ratings of the negative, neutral and positive monetary and social outcomes in control and low emotional apathy (LEA) and high emotional apathy (HEA) patient subgroups^1^

Outcome type	Control	LEA	HEA	Group difference (*p* value)	*Post hoc* group comparisons
Social negative	1.69 ± 0.68	1.76 ± 0.67	2.34 ± 0.86	0.005	Con, LEA < HEA
Social neutral	3.63 ± 0.54	3.64 ± 0.56	3.80 ± 0.61	0.858	-
Social positive	6.20 ± 1.05	6.38 ± 1.94	5.65 ± 1.31	0.335	-
Monetary negative	1.89 ± 0.89	1.94 ± 1.01	2.04 ± 1.43	0.983	-
Monetary neutral	4.04 ± 1.02	3.78 ± 1.21	3.52 ± 1.37	0.693	-
Monetary positive	6.04 ± 1.40	6.44 ± 0.91	6.11 ± 1.45	0.483	-

^1^Values are mean ± standard deviation.

#### Relationships between emotion perception, social and monetary reward learning, and emotional apathy

Linear regression analysis was conducted to examine whether emotion perception performance or social and monetary learning performance (choice test scores) predicted emotional apathy in dementia patients. Patient diagnosis, disease duration, and general cognition (ACE-III) were also entered into the initial model. As shown in Table 3, the final model was significant (*F*(3,47) = 10.406, *p* < 0.001) and explained 39.9% of the total variance in emotional apathy (Table 3). In the final model, a diagnosis of behavioural-variant frontotemporal dementia (relative to Alzheimer’s disease) predicted *higher* emotional apathy (*ß* = 0.399, *t* = 3.361, *p* = 0.002), whereas greater social reward learning significantly predicted *lower* emotional apathy (*ß* = –0.255, *t* = –1.766, *p* = 0.045). Monetary reward learning remained in the final model but was not a significant predictor (*ß* = –0.228, *t* = 2.056, *p* = 0.084). Emotion perception performance did not significantly predict emotional apathy and was removed from the final model.

**Table 3:** Variables contributing to emotional, executive and initiation apathy in the final regression models

	B	SE B	*ß*	*T*	*p*
Emotional apathy					
(Constant)	18.052	2.21		8.168	<0.001
bvFTD diagnosis	4.376	1.302	0.399	3.361	0.002
Monetary reward learning	−4.68	2.65	−0.228	−1.766	0.084
Social reward learning	−5.464	2.658	−0.255	−2.056	0.045
Executive apathy					
(Constant)	30.085	3.745		8.224	<0.001
bvFTD diagnosis	5.865	1.248	0.508	4.700	<0.001
LPA diagnosis	−6.828	2.666	−0.252	−2.561	0.014
ACE-III	−0.188	0.055	−0.393	−3.444	0.001
Social reward learning	−8.193	2.363	−0.363	−3.467	0.001
Initiation apathy					
(Constant)	27.710	4.208		6.586	<0.001
bvFTD diagnosis	4.644	1.323	0.473	3.511	0.001
SD diagnosis	–3.936	1.987	–0.265	–1.981	0.053
ACE-III	–0.182	0.055	–0.447	–3.338	0.002

**R**^**2**^ **and R changes between models for emotional apathy**: Model 1: R^2^ = 0.440, *p* = 0.002; Model 2: R^2^ = 0.440, *p* = 0.001; ΔR^2^ = 0, *p* = 0.951; Model 3: R^2^ = 0.438, *p* < 0.001; ΔR^2^ = −0.002, *p* = 0.725; Model 4: R^2^ = 0.435, *p* < 0.001; ΔR^2^ = −0.003, *p* = 0.628; Model 5: R^2^ = 0.429, *p* < 0.001; ΔR^2^ = −0.006, *p* = 0.496; Model 6: R^2^ = 0.416, *p* < 0.001; ΔR^2^ = −0.013, *p* = 0.318; Model 7 (final): R^2^ = 0.399, *p* < 0.001; ΔR^2^ = −0.017, *p* = 0.250.

**R**^**2**^ **and R changes between models for executive apathy:** Model 1: R^2^ = 0.623, *p* < 0.001; Model 2: R^2^ = 0.621, *p* < 0.001; ΔR^2^ = −0.001, *p* = 0.732; Model 3: R^2^ = 0.616, *p* < 0.001; ΔR^2^ = −0.006, *p* = 0.438; Model 4: R^2^ = 0.609, *p* < 0.001; ΔR^2^ = −0.007, *p* = 0.394; Model 5: R^2^ = 0.604, *p* < 0.001; ΔR^2^ = −0.006, *p* = 0.426; Model 6 (final): R^2^ = 0.590, *p* < 0.001; ΔR^2^ = −0.014, *p* = 0.215.

**R**^**2**^ **and R changes between models for initiation apathy:** Model 1: R^2^ = 0.418, *p* = 0.004; Model 2: R^2^ = 0.417, *p* = 0.002; ΔR^2^ = −0.001, *p* = 0.782; Model 3: R^2^ = 0.415, *p* = 0.001; ΔR^2^ = −0.002, *p* = 0.706; Model 4 : R^2^ = 0.408, *p* < 0.001; ΔR^2^ = −0.007, *p* = 0.480; Model 5: R^2^ = 0.387, *p* < 0.001; ΔR^2^ = −0.020, *p* = 0.224; Model 6: R^2^ = 0.367, *p* < 0.001; ΔR^2^ = −0.020, *p* = 0.232; Model 7 (final): R^2^ = 0.336, *p* < 0.001; ΔR^2^ = −0.031, *p* = 0.141.

SE B = standard error of B; bvFTD = behavioural-variant frontotemporal dementia; LPA = logopenic progressive aphasia; SD = semantic dementia; ACE-III = Addenbrooke’s Cognitive Examination, 3^rd^ Edition.

To clarify whether such relationships between monetary and social reward learning and emotional apathy are specific to emotional apathy, additional regression analyses were performed using the same predictor variables for executive apathy and initiation apathy scores from the DAS (Table 3). For executive apathy, social reward learning, a diagnosis of behavioural-variant frontotemporal dementia or logopenic progressive aphasia (relative to Alzheimer’s disease), and general cognition (ACE-III) were significant predictors and explained 59.0% of the total variance (*F*(4,46) = 16.530, *p* < 0.001). Neither social nor monetary reward learning predicted initiation apathy, where the final model indicated that a diagnosis of behavioural-variant frontotemporal dementia or semantic dementia (relative to Alzheimer’s disease) and ACE-III scores explained 33.6% of the total variance in initiation apathy (*F*(2,47) = 7.942, *p* < 0.001). To summarise, social reward learning was an important predictor of both emotional and executive apathy but did not appear to be involved in the manifestation of initiation apathy.

### Neuroimaging

#### Group differences in grey matter intensity

Profiles of grey matter intensity decrease in the LEA and HEA groups relative to controls are reported in Supplementary Figure 2 and Supplementary Table 2. Compared with controls, LEA patients presented with extensive bilateral atrophy across frontal, temporal, insular, striatal, parietal, and occipital regions. Relative to controls, HEA patients also showed widespread bilateral atrophy across largely similar regions. No significant regions of grey matter intensity decrease were observed in the HEA group relative to LEA group, or vice versa at *p* < 0.005 with Family-Wise Error correction. At the *p* < 0.005 uncorrected threshold, however, the HEA group showed greater atrophy in the right striatum and thalamus and left postcentral/precentral gyrus relative to LEA patients, whereas the LEA group showed greater atrophy in medial temporal and occipital-temporal regions relative to HEA patients (Supplementary Figure 2; Supplementary Table 2).

#### Shared grey matter correlates of emotional apathy and social reward learning

**Fig. 3:**
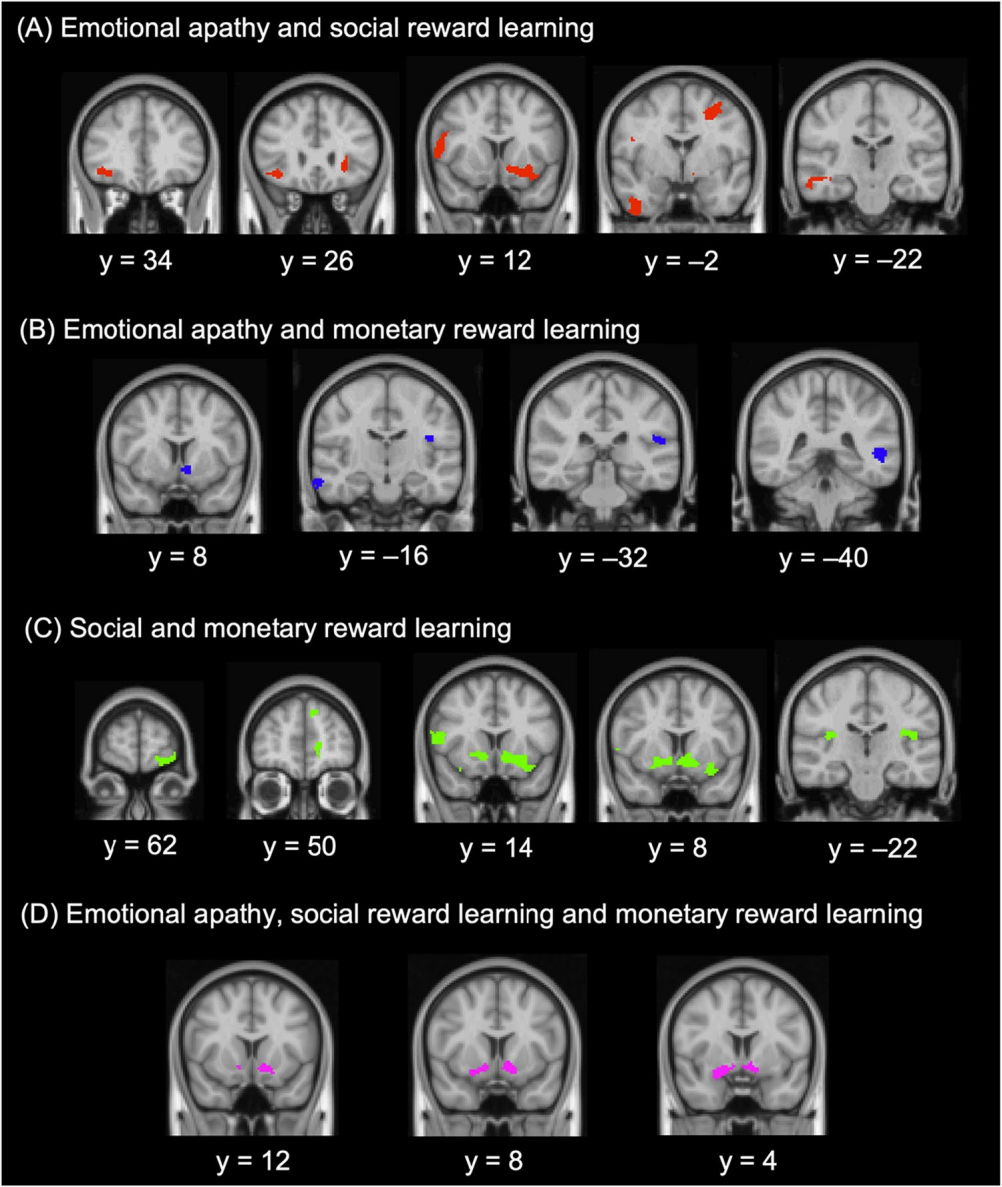
Voxel-based morphometry results. Regions of grey matter intensity decrease common to **(A)** emotional apathy and social reward learning, **(B)** emotional apathy and monetary reward learning, **(C)** social reward learning and monetary reward learning, and **(D)** emotional apathy, social reward learning, and monetary reward learning. Coloured voxels indicate regions that emerged as significant in the voxel-based morphometry analyses, corrected for false discovery rate at *p* < 0.05. All clusters reported *t* > 2.68. Clusters are overlaid on the Montreal Neurological Institute (MNI) standard brain. Numbers are MNI coordinates for coronal sections. R = right; L = left

As shown in Figure 3A and Table 4, a set of fronto-striatal-insular regions were jointly implicated in both emotional apathy and social reward learning, including the orbitofrontal and lateral prefrontal (inferior and middle frontal gyri) cortices bilaterally, as well as the left striatum (nucleus accumbens and putamen), left insular cortex, and right frontopolar cortex, inferior, and middle temporal gyri.

**Table 4:** Regions of significant grey matter intensity decrease common to (**a**) emotional apathy and social reward learning, (**b**) emotional apathy and monetary reward learning, (**c**) social reward learning and monetary reward learning, and (**d**) emotional apathy, social reward learning and monetary reward learning

Regions		Peak MNI coordinates			
	Hemisphere	X	Y	Z	Cluster size
***(a) Emotional apathy and social reward learning***					
Orbitofrontal cortex, nucleus accumbens, putamen, insular cortex	L	−22	14	−14	542
Inferior frontal gyrus	R	58	10	2	288
Middle frontal gyrus	L	−26	−2	46	228
Inferior temporal gyrus (anterior)	R	42	0	−40	198
Orbitofrontal cortex, frontal pole	R	40	34	−8	160
Middle temporal gyrus (posterior)	R	58	−18	−20	111
***(b) Emotional apathy and monetary reward learning***					
Nucleus accumbens	L	−2	2	−12	226
Middle temporal gyrus (posterior)	R	70	−16	−24	162
Middle temporal gyrus (posterior)	L	−44	−40	2	123
Superior temporal gyrus (planum temporale)	L	−54	−32	16	109
***(c) Social reward learning and monetary reward learning***					
Nucleus accumbens, putamen, caudate (left), orbitofrontal cortex (left), insular cortex (left)	B	0	4	−14	1143
Inferior frontal gyrus	R	40	18	16	258
Central opercular cortex	L	−36	−18	20	187
Frontal pole	L	−16	56	−6	186
Anterior cingulate gyrus	B	6	−10	26	183
Parietal operculum cortex, insular cortex	R	36	−24	16	157
Superior frontal gyrus	L	−12	36	38	157
Occipital pole	R	26	−96	−10	142
***(d) Emotional apathy, social reward learning and monetary reward learning***					
Nucleus accumbens	L	−12	6	−12	177
Amygdala, orbitofrontal cortex, nucleus accumbens	R	24	2	−6	168

Clusters were extracted voxel-wise corrected for false discovery rate at *p* < 0.05. All clusters reported t > 2.68, with a cluster extent threshold of >100 contiguous voxels.

L = left; R = right; B = bilateral; MNI = Montreal Neurological Institute.

#### Shared grey matter correlates of emotional apathy and monetary reward learning

Figure 3B and Table 4 display the results from the voxel-based morphometry analysis exploring associations between severity of grey matter intensity decrease and both emotional apathy and monetary reward learning across the entire patient cohort. The left nucleus accumbens was implicated as a shared region of significant grey matter intensity decrease, together with regions in the left superior temporal gyrus (planum temporale) and bilateral posterior middle temporal gyri.

#### Shared grey matter correlates of social reward learning and monetary reward learning

Figure 3C and Table 4 show regions of grey matter intensity decrease that were common to reward learning performance across both the monetary and social conditions. Striatal regions were implicated, including the bilateral nucleus accumbens and putamen, extending into the left caudate and orbitofrontal and insular cortices, as well as the right insular cortex and inferior frontal gyrus. In addition, reward learning across both conditions was associated with grey matter intensity decrease in the anterior cingulate cortex bilaterally, together with regions in the left fronto-polar and superior frontal gyri and TPJ (central opercular cortex). Ancillary regions in the right parietal operculum cortex and occipital pole were also involved.

#### Shared grey matter correlates of emotional apathy, social reward learning and monetary reward learning

Figure 3D and Table 4 show regions of grey matter intensity decrease that were common to emotional apathy as well as reward learning performance across both the monetary and social conditions. Again, striatal regions were implicated, including the bilateral nucleus accumbens, extending into the orbitofrontal cortex and amygdala on the right.

## Discussion

This study examined the mechanisms underlying emotional apathy in dementia and aimed to establish whether emotional apathy and social reward learning have a shared neurobiological basis. Importantly, our study is the first to apply a transdiagnostic approach to investigate these symptoms and mechanisms across patients with different subtypes of dementia, who were classified into high versus low emotional apathy subgroups. Our analyses revealed that patients with high levels of emotional apathy show impaired learning from both social and monetary rewards, as well as impaired emotion perception. In contrast, those with lower levels of emotional apathy demonstrated relatively intact learning from social and monetary rewards during the choice test, together with only mild emotion perception difficulties. Importantly, we found that social reward learning, but not emotion perception, predicted greater emotional apathy across all patients. Our voxel-based morphometry analyses revealed shared neural correlates in the orbitofrontal cortex, striatum and insula, indicating that emotional apathy and social reward learning are associated with degeneration in key reward processing regions of the brain. The theoretical and clinical implications of these findings are discussed in the following sections.

According to multidimensional models of apathy, emotional apathy is purportedly underpinned by an inability to associate socio-emotional signals with behaviour, which is important for guiding and evaluating behaviour in terms of their positive and negative outcomes (Levy and Dubois, 2006). Importantly, this is the first study to empirically validate this claim by demonstrating that patients with high levels of emotional apathy show impaired reward learning from socially positive and negative outcomes. Moreover, we found that social reward learning performance predicted the severity of emotional apathy in our cohort of dementia patients. On the question of whether emotional apathy reflects a *general* impairment in reward learning, or whether it is specific to social rewards, we found that although patients with high emotional apathy showed impaired learning from both social and monetary rewards, the latter appeared to play a relatively weaker role in predicting emotional apathy. Across all patients, social and monetary reward learning exhibited similar effect sizes for predicting emotional apathy, but the effect of monetary reward learning was only significant at trend level. While our results align with previous work reporting reduced sensitivity to monetary rewards in apathetic behavioural-variant frontotemporal dementia patients (Massimo, 2015), this is the first study to directly contrast social and monetary reward learning in these patients. Critically, our results extend these findings by demonstrating that reward learning deficits are also observed for socially positive and negative outcomes, and that symptoms of emotional apathy are more closely linked to deficits in using these social cues to guide behaviour. Of relevance, deficits in integrating social contextual information to modulate complex social behaviours, such as prosociality and punishment (O'Callaghan et al., 2016) and vulnerability to financial exploitation (Wong et al., 2017), have previously been identified in behavioural-variant frontotemporal dementia. Nonetheless, our study is the first to link social reward learning deficits to symptoms of emotional apathy more broadly across different dementia syndromes.

Considering emotion perception next, we did not find a significant predictive relationship between emotion perception performance and severity of emotional apathy in our dementia cohort. This was despite the finding that both patient groups demonstrated poorer emotion perception relative to controls, with those in the high emotional apathy group showing the greatest impairment. On the question of whether the underlying mechanism of emotional apathy involves a breakdown in the *perception* of socioemotional rewards, or deficits in the *use* of such rewards to modify behaviour, our results therefore support the latter. Interestingly, we also found that subjective ratings of some reward outcomes were altered in patients with high emotional apathy, such that they rated socially negative outcomes as less “unpleasant” compared with controls and patients with low emotional apathy. This is consistent with previous studies in behavioural-variant frontotemporal dementia patients, who rated emotional stimuli as less strongly valenced (for both positive and negative emotions) than controls (Kumfor et al., 2019). Similarly, reduced sensitivity to negative outcomes, such as losing money (Massimo, 2015; Perry et al., 2015) or unpleasant odours (Perry et al., 2017) have also been demonstrated in behavioural-variant frontotemporal dementia. While our social reward learning paradigm encompassed both positive and negative social outcomes, it is unclear how perceptions or experiences of outcome valence impact on the link between reward learning and emotional apathy. Furthermore, recent evidence points to clinically significant levels of anhedonia in behavioural-variant frontotemporal dementia and semantic dementia (Shaw et al., 2021), which may also potentially impact on reward learning. As such, the relationships between anhedonia, reward learning, and emotional apathy will be an important area for future studies.

Another significant predictor of emotional apathy was dementia subtype. Specifically, our regression analysis revealed that a diagnosis of behavioural-variant frontotemporal dementia (as opposed to a diagnosis of Alzheimer’s disease) was associated with greater symptoms of emotional apathy. This is in line with previous studies showing milder emotional apathy in the early stages of Alzheimer’s disease compared with behavioural-variant frontotemporal dementia (Wei et al., 2019). Nonetheless, a proportion of Alzheimer’s disease (14%), semantic dementia (38%), progressive nonfluent aphasia (17%), and logopenic progressive aphasia (33%) patients in our cohort had clinically significant symptoms of emotional apathy based on established cutoff criteria (Radakovic et al., 2016). Likewise, 36% of the behavioural-variant frontotemporal dementia patients in our cohort were classified into the low emotional apathy group, which did not differ from controls on the social and monetary reward learning task. From a clinical perspective, the heterogeneity in presentation of emotional apathy symptoms and reward learning deficits further emphasises the utility in adopting a more nuanced, transdiagnostic approach to the assessment and management of emotional apathy, as opposed to approaches that purely focus on clinical diagnosis. While the majority of behavioural-variant frontotemporal dementia patients present with high levels of emotional apathy, these symptoms may remain relatively mild for a significant proportion of patients, as demonstrated in our sample. Of note, previous reports of significant heterogeneity in phenotypic presentations and patterns of functional network degeneration in behavioural-variant frontotemporal dementia (Ranasinghe et al., 2016) also support the need for a shift towards symptom-driven, rather than diagnosis-driven, approaches. This approach is especially important in syndromes, such as semantic dementia, progressive nonfluent aphasia, and logopenic progressive aphasia, where symptoms of apathy are common but may be overlooked in the context of more prominent language symptoms (Wong et al., 2020). Disease-specific approaches may potentially overlook behavioural symptoms, such as apathy. Given that apathy is a key determinant of carer burden across various dementia syndromes (Armstrong et al., 2013; Liu et al., 2016; Wong et al., 2020), raising awareness and understanding of these symptoms and developing tailored interventions will be critical areas of future inquiry.

Turning to our neuroimaging findings, our analyses revealed a set of shared brain regions for emotional apathy and social reward learning, specifically the bilateral orbitofrontal cortex, left ventral striatum and left insular cortex, as well as ancillary regions in the lateral prefrontal cortices bilaterally and right lateral temporal cortices. Notably, our results demonstrate for the first time that emotional apathy and social reward learning are both associated with atrophy in key regions in the brain’s reward circuitry. This finding resonates with two parallel bodies of literature, which have identified the orbitofrontal cortex and ventral striatum to be critical for emotional apathy (Levy and Dubois, 2006; Radakovic and Abrahams, 2018) and reward learning (O'Doherty, 2004; Rolls, 2000). This study demonstrates that these two constructs converge on the neural level. The orbitofrontal cortex plays a critical role in signalling the emotional value of perceived stimuli and encoding reward expectations (O'Doherty, 2007; Schoenbaum and Roesch, 2005), which are integrated by other prefrontal regions (e.g., dorsolateral prefrontal cortex) to guide goal-directed behaviour (Wallis, 2007). Our findings therefore suggest that disruption to the capacity of socioemotional rewards to drive behaviour may account for symptoms of emotional apathy, such as emotional blunting, reduced empathy, and diminished social interactions. Likewise, the ventral striatum is implicated in associative learning and motivation (Husain and Roiser, 2018; Liljeholm and O'Doherty, 2012), via its role in processing reward prediction errors and reward-based learning (Knutson et al., 2001; O'Doherty et al., 2004). As a shared neural substrate of both emotional apathy and social reward learning, degeneration of the ventral striatum likely disrupts the ability to learn and modify behaviour in accordance with social outcomes.

The insular cortex was also implicated in emotional apathy and social reward learning. Although insular involvement was not originally proposed by Levy and Dubois (2006), this region is increasingly recognised as a critical hub for interoception, emotion and social cognition (Adolfi et al., 2017; Van den Stock and Kumfor, 2019). Degeneration of the insula has also been linked with emotional apathy, as well as changes in social reward learning, social cognition and empathic concern (Couto et al., 2013; Dermody et al., 2016; Quang et al., 2021; Wei et al., 2019; Wong et al., 2017). Notably, insula atrophy has been implicated in reduced physiological responses and facial expressivity to emotional stimuli in frontotemporal dementia (Kumfor et al., 2019). It also plays a central role in the Salience Network, by directing attention to salient internal and external stimuli to guide behaviour (Kumfor et al., 2015; Menon and Uddin, 2010). As such, deficits in integrating physiological and interpersonal socioemotional signals with purposeful behaviour may disrupt learning from social rewards, which may in turn, manifest as emotional apathy.

On the question of whether greater neuroanatomical convergence exists between emotional apathy and social compared with monetary reward learning, a relatively restricted set of brain regions was jointly implicated in emotional apathy and monetary reward learning, including the ventral striatum, left superior temporal gyrus, and bilateral posterior middle temporal gyri. The ventral striatal overlap between emotional apathy and monetary reward learning converges well with this region’s fundamental role in reward-based learning (Knutson et al., 2001; O'Doherty et al., 2004). Importantly, this role does not appear to vary according to reward type (Daniel and Pollmann, 2014; Lin, Adolphs, et al., 2012a). The robust association between ventral striatal atrophy and both social and monetary reward learning, and its shared role across emotional apathy as well as social and monetary reward learning, also meshes well with our behavioural findings and suggests a general reward learning deficit in these patients. Taken together, our behavioural and neuroimaging findings therefore indicate that emotional apathy is associated with disruption to fundamental mechanisms of reward learning, as well as changes in the neurocognitive mechanisms that integrate socioemotional signals to drive socially and emotionally relevant behaviour. An important caveat to these neuroimaging results, however, is that we did not identify significant differences in overall patterns of atrophy between patients with low versus high emotional apathy. While some differences were observed at uncorrected thresholds, future studies that apply a more targeted regional approach (e.g., focusing on fronto-striatal-insular regions rather than whole-brain analysis) with larger patient cohorts may yield more consistent results.

Unexpectedly, social reward learning together with general cognitive impairment also predicted executive apathy. While this may potentially be related to deficits in the strategic learning aspects of the reward learning task, it is unclear why social, but not monetary reward learning predicted executive apathy. Future studies that employ reward processing paradigms with less reliance on learning and memory may be helpful to distinguish between the contributions of socioemotional reward sensitivity and strategic learning and memory to executive apathy. In addition to this, some methodological limitations warrant consideration. Firstly, the reward learning paradigm did not assess emotion perception and social reward learning using the same stimuli. While participants were asked to provide subjective ratings of the pleasantness of each of the social outcomes at the end of the learning task, we did not ask them to identify the emotional expression shown in each outcome. Future studies that incorporate emotion perception tests using the same stimuli may therefore enable more fine-grained contrast of the influence of emotion perception and reward learning on emotional apathy. Studies that employ concurrent physiological measures of arousal may also be of value, given the potential mismatch between participants’ subjective ratings and objective reward responsivity or ability to perceive positive outcomes as inherently rewarding (Balconi et al., 2015) and the potential relationship with interoception. Indeed, while altered physiological responsivity to emotional stimuli has been reported in frontotemporal dementia (Kumfor et al., 2019), whether this disturbance impacts on reward learning and emotional apathy remains to be explored. Furthermore, although our low and high emotional apathy patient groups were classified based on established cut-off scores for clinically significant emotional apathy (Radakovic et al., 2016), this methodological approach may potentially underestimate group differences, particularly for patients scoring near the cutoff. Although this approach has clear clinical utility, future studies contrasting patients from either end of the emotional apathy severity scale may provide further insights into the neurobiological mechanisms underlying this symptom. Additionally, some dimensional models of apathy further distinguish between emotional and social aspects of apathy, such that emotional sensitivity relates to feelings of positive and negative affection, whereas social motivation refers to an individual’s engagement in social interactions (Ang et al., 2017). Given that these aspects of apathy could not be disentangled in the current study, future research using measures such as the Apathy Motivation Index (Ang et al., 2017) would be of interest to clarify the impact of social reward learning impairment on emotional versus social aspects of apathy. Finally, given that our cohort of patients had relatively similar linguistic and educational backgrounds, the generalisability of our findings to more diverse patient populations remains to be established. Future studies that include cross-cultural comparisons or control for race/ethnicity may therefore be of value to determine whether these factors influence the relationship between social reward learning and emotional apathy.

From a clinical perspective, our findings may inform treatment options. Thus far, pharmacological and nonpharmacological interventions have shown limited efficacy for managing apathy (Berman et al., 2012; Brodaty and Burns, 2012). This may partly be due to previous conceptualisations of apathy as a unitary construct. Our results indicate that interventions that focus on supporting deficits in socioemotional reward processing may prove beneficial for alleviating symptoms of emotional apathy. One promising approach is behavioural activation therapy, a well-established treatment for depression, which aims to increase an individual’s engagement in pleasurable, productive, or personally meaningful activities that foster positive reinforcement (Richards et al., 2016). By focusing on reward-directed behaviour, this approach may potentiate reward-related networks in the brain via improvements in reward anticipation, responsivity, and learning (Nagy et al., 2020). Nonetheless, further research is needed to clarify the underlying neural mechanisms of behavioural activation therapy and to determine its effectiveness for symptoms of apathy in individuals with dementia. Likewise, interventions that seek to support processing of interoceptive and physiological markers of reward may help to boost an individual’s reward processing ability; however, further research establishing the link between such markers and symptoms of emotional apathy is needed.

In summary, this study demonstrates that emotional apathy is prevalent in a substantial proportion of dementia patients, and those with high levels of emotional apathy show widespread impairments in reward learning. Our results provide the first empirical evidence that emotional apathy is underpinned by deficits in the ability to use socioemotional cues to guide goal-directed behaviour, and the degeneration of brain regions responsible for reward processing, particularly for socially positive and negative outcomes. Future development of interventions that take a more nuanced approach to target these underlying mechanisms will help increase patient engagement and improve the quality of life of patients and families.

## Supplementary Information

ESM 1 (PDF 3939 kb)

## Data Availability

The conditions of our ethics approval do not permit public archiving of anonymised study data. Readers seeking access to the data should contact the corresponding author. Access will be granted to named individuals in accordance with ethical procedures governing the reuse of clinical data, including completion of a formal data sharing agreement and approval of the local ethics committee. This study was not pre-registered.

## Abbreviations

ACE-III: Addenbrooke’s Cognitive Examination, 3^rd^ Edition
DAS: Dimensional Apathy Scale
HEA: high emotional apathy
LEA: low emotional apathy
