# Supplementary material for: Altered reward processing underpins emotional apathy in dementia
Source: Cogn Affect Behav Neurosci. 2022 Nov 23;23(2):354–70. doi: 10.3758/s13415-022-01048-2 (PMC10049956; doi:10.3758/s13415-022-01048-2)
Supplement: Supplementary file 1 — (PDF 3939 kb) [file 13415_2022_1048_MOESM1_ESM.pdf]

**Supplementary Materials**

***Supplementary Table 1.*** Demographic and clinical characteristics of diagnostic groups.

| | Control | bvFTD | AD | SD | PNFA | LPA | Group difference (*p*-value) | *Post hoc* group comparisons |
|---|---|---|---|---|---|---|---|---|
| N | 27 | 33 | 14 | 8 | 6 | 3 | | |
| Sex (M:F) | 10:17 | 26:7 | 9:5 | 5:3 | 2:4 | 3:0 | .012 | |
| Age (years) | 65.99 ± 7.62 | 63.23 ± 8.35 | 65.44 ± 8.88 | 62.38 ± 4.64 | 65.24 ± 9.38 | 69.56 ± 4.20 | .409 | |
| Education (years) | 13.40 ± 2.72 | 11.67 ± 3.87 | 13.89 ± 2.87 | 13.06 ± 2.57 | 11.46 ± 1.89 | 11.58 ± 2.50 | .078 | |
| Disease duration (years) | . | 6.65 ± 4.87 | 4.25 ± 1.07 | 4.52 ± 1.60 | 5.57 ± 3.97 | 2.03 ± 1.11 | .146 | |
| FRS Rasch score | . | -0.33 ± 1.37 | 0.74 ± 1.26 | 1.34 ± 1.50 | 3.39 ± 1.16 | 2.53 ± 0.47 | <.001 | bvFTD < SD, PNFA, LPA; AD < PNFA |
| ACE-III total [100] | 94.30 ± 3.26 | 82.03 ± 9.93 | 71.00 ± 11.94 | 69.00 ± 12.69 | 85.00 ± 6.99 | 64.67 ± 11.24 | <.001 | Con > bvFTD, AD, SD, LPA |
| DASS depression [42] | 1.04 ± 1.29 | 10.2 ± 9.42 | 6.43 ± 6.28 | 8.57 ± 7.37 | 8.00 ± 8.85 | 5.33 ± 6.11 | <.001 | Con < bvFTD |
| DASS anxiety [42] | 1.26 ± 1.85 | 8.07 ± 6.18 | 4.46 ± 6.59 | 3.14 ± 3.44 | 6.33 ± 4.97 | 2.67 ± 4.62 | <.001 | Con < bvFTD |
| DASS stress [42] | 3.93 ± 4.69 | 12.2 ± 10.31 | 9.29 ± 7.75 | 7.00 ± 5.33 | 8.67 ± 6.02 | 8.67 ± 9.02 | .012 | Con < bvFTD |
| DAS total [72] | 18.04 ± 10.33 | 50.39 ± 11.21 | 40.86 ± 10.08 | 35.38 ± 16.02 | 26.50 ± 8.38 | 32.33 ± 13.80 | <.001 | Con < bvFTD, AD; PNFA < bvFTD |
| DAS executive [24] | 3.68 ± 3.80 | 16.45 ± 5.32 | 14.07 ± 4.38 | 10.75 ± 5.42 | 7.33 ± 4.03 | 7.33 ± 1.53 | <.001 | Con < bvFTD, AD; PNFA < bvFTD |
| DAS emotional [24] | 7.16 ± 4.01 | 16.52 ± 4.84 | 11.64 ± 3.46 | 12.50 ± 6.57 | 7.17 ± 4.12 | 12.67 ± 4.73 | <.001 | Con < bvFTD; PNFA < bvFTD |
| DAS initiation [24] | 7.20 ± 5.13 | 17.42 ± 4.24 | 15.14 ± 3.94 | 12.13 ± 6.03 | 12.00 ± 3.79 | 12.33 ± 7.77 | <.001 | Con < bvFTD, AD; PNFA < bvFTD |
| Emotional apathy group (LEA:HEA) | . | 12:21 | 12:2 | 5:3 | 5:1 | 2:1 | .015 | |
| Emotion perception [42] | 39.00 ± 3.06 | 32.38 ± 5.72 | 32.17 ± 4.71 | 34.50 ± 4.87 | 36.00 ± 5.15 | 34.0 ± 5.66 | <.001 | Con < bvFTD, AD |

Values are mean ± standard deviation. Maximum scores on each measure shown in brackets. FRS = Frontotemporal dementia Rating Scale; ACE-III = Addenbrooke's Cognitive Examination, Third Edition; DASS = Depression, Anxiety and Stress Scale; DAS = Dimensional Apathy Scale
Missing data: DAS (3 controls); Emotion perception (2 Controls, 4 bvFTD, 2 AD, 1 PNFA, 1 LPA)

***Supplementary Figure 1.*** Overall social and monetary reward learning performance, denoted by proportion of optimal choices made in extinction on the post-learning choice test for the social and monetary conditions across the control, LEA and HEA groups. Data points indicate individual participant scores, with colours denoting diagnosis group. Error bars represent standard error of the mean.

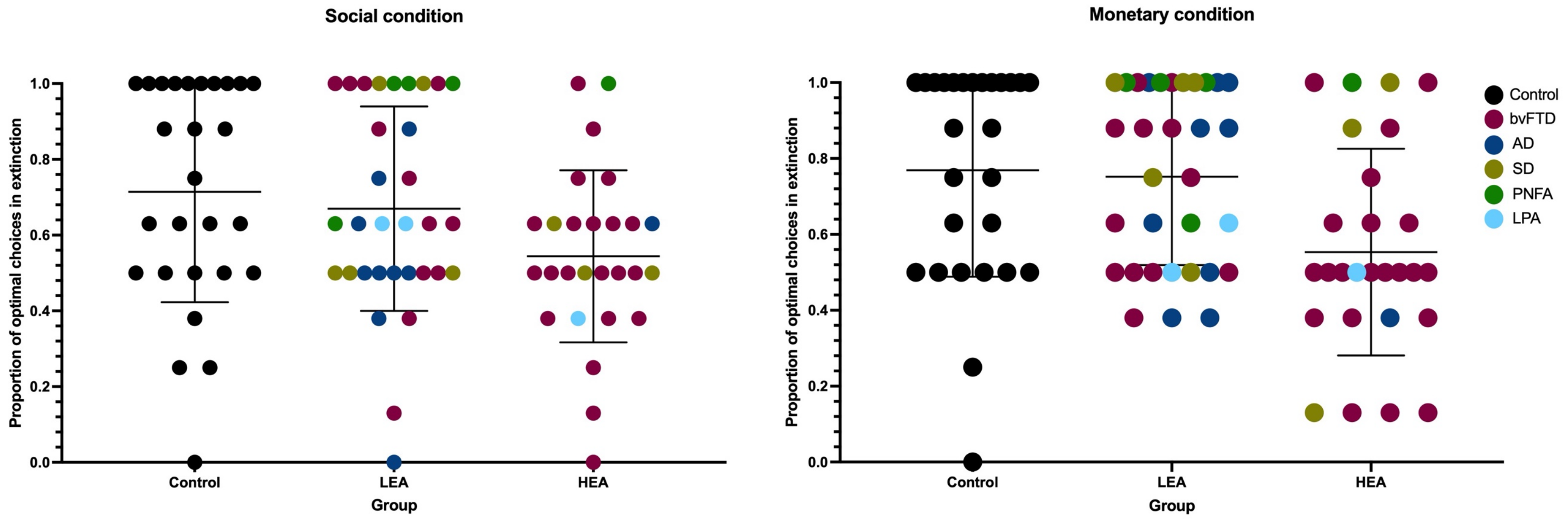

***Supplementary Figure 2.*** Regions of significant grey matter intensity decrease in (A) LEA and (B) HEA patient groups relative to controls (C) LEA relative to HEA patients and (D) HEA relative to LEA patients. Coloured voxels indicate regions that emerged as significant in the voxel-based morphometry analyses, at $p$<.005 corrected for family-wise error in (A) and (B) and $p$<.005 uncorrected for (C) and (D). All clusters reported at t > 2.660. Clusters are overlaid on the Montreal Neurological Institute (MNI) standard brain. Numbers are MNI coordinates for coronal sections. R = right; L = left.

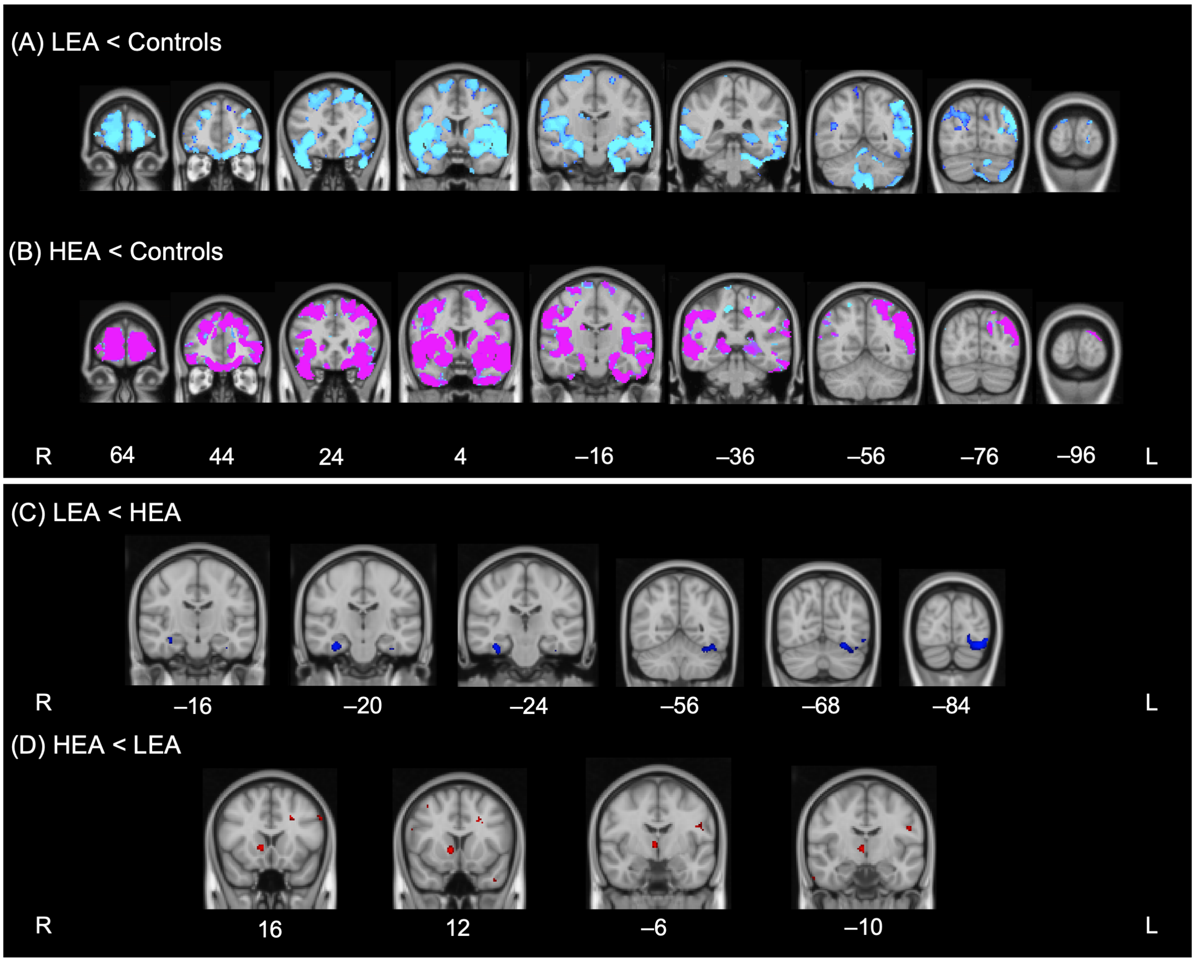

***Supplementary Table 2.*** Group differences in grey matter intensity across LEA and HEA groups

| Contrast | Regions | Hemisphere | Peak MNI coordinates | | | Cluster size |
|---|---|---|---|---|---|---|
| | | | X | Y | Z | |
| LEA < Controls | Cerebellum, fusiform cortex, inferior, middle and superior temporal gyri, hippocampus, amygdala, temporal pole, caudate, putamen, insular cortex, orbitofrontal cortex, frontal medial cortex, paracingulate cortex, anterior cingulate cortex, frontal pole, inferior middle and superior frontal gyri, supramarginal gyrus, angular gyrus, lateral occipital cortex, planum temporale, central and parietal opercular cortex | L | -8 | -56 | -64 | 35568 |
| | Temporal pole, orbitofrontal cortex, insular cortex, putamen, caudate, thalamus, amygdala, hippocampus, inferior and middle frontal gyri, inferior, middle and superior temporal gyri, Heschl's gyrus, planum temporal, central and parietal opercular cortex, supramarginal gyrus | R | 40 | 20 | -42 | 11913 |
| | Lateral occipital cortex (superior), cuneal cortex | R | 24 | -84 | 34 | 1537 |
| | Precuneus cortex | R | 8 | -48 | 64 | 274 |
| | Superior frontal gyrus | L | -6 | 52 | 38 | 214 |
| HEA < Controls | Fusiform gyrus, inferior temporal gyrus, middle temporal gyrus, superior temporal gyrus, insular cortex, amygdala, hippocampus, paracingulate cortex, anterior cingulate cortex, orbitofrontal cortex, medial frontal cortex, frontal pole, inferior frontal gyrus, middle frontal gyrus, superior frontal gyrus, precentral gyrus, postcentral gyrus, supramarginal gyrus, precuneus, lateral occipital cortex, occipital pole, caudate, putamen, thalamus | B | 30 | -6 | -52 | 61458 |
| | Precentral gyrus | R | 14 | -16 | 76 | 258 |
| LEA < HEA* | Lateral occipital cortex, occipital fusiform cortex, temporal-occipital fusiform cortex | L | -34 | -84 | -20 | 927 |
| | Temporal fusiform cortex, parahippocampal gyrus, hippocampus | R | 34 | -22 | 26 | 127 |
| HEA < LEA* | Caudate, nucleus accumbens | R | 12 | 12 | 9 | 66 |
| | Postcentral gyrus, precentral gyrus | L | -48 | -12 | 26 | 58 |
| | Thalamus | R | 42 | 58 | 37 | 55 |

*Clusters were Family Wise Error corrected at p<.005 and at a cluster extent threshold of >200 contiguous voxels.*
**Clusters were uncorrected at p<.005 and at a cluster extent threshold of >50 contiguous voxels.*
*L = left; R = right; B = bilateral; MNI = Montreal Neurological Institute.*

## Grey matter correlates of emotional apathy

Supplementary Figure 2 and Table 3 display the results from the voxel-based morphometry analysis exploring associations between regions of grey matter intensity decrease and emotional apathy on the DAS across all patients. Higher levels of emotional apathy were associated with grey matter intensity decrease across the left insular cortex, orbitofrontal cortex and lateral occipital cortex, as well as the right striatum (including nucleus accumbens, caudate and putamen), postcentral gyrus and cerebellum. Bilateral regions in the supramarginal gyri were also implicated.

## Grey matter correlates of social reward learning

As shown in Supplementary Figure 2 and Table 3, lower scores on the choice test in the social condition, reflecting poorer learning from social rewards, were associated with grey matter intensity decrease in the orbitofrontal and insular cortices and striatum (nucleus accumbens, caudate and putamen) bilaterally. Furthermore, regions in the left lateral prefrontal cortex (middle and superior frontal gyri, extending into supplementary motor cortex) and the right inferior frontal gyrus (extending into the precentral gyrus) and inferior temporal gyrus were also implicated.

## Grey matter correlates of monetary reward learning

As shown in Supplementary Figure 2 and Table 3, lower scores on the choice test in the monetary condition, reflecting poorer learning from monetary rewards, were associated with grey matter intensity decrease in the nucleus accumbens, subcallosal cortex and anterior cingulate bilaterally, as well as the right insular cortex, extending into Heschl’s gyrus and parietal operculum cortex, and the right forntal pole, lateral prefrontal cortex (inferior and middle frontal gyri) and middle temporal gyrus. Regions in the left central and parietal opercular cortices were also involved.

***Supplementary Figure 3.*** Grey matter correlates of (a) emotional apathy, (b) social reward learning and (c) monetary reward learning across all patients. Coloured voxels indicate regions that emerged as significant in the voxel-based morphometry analyses, corrected for false discovery rate at $p<.05$. All clusters reported $t > 2.63$. Clusters are overlaid on the Montreal Neurological Institute (MNI) standard brain. Numbers are MNI coordinates for coronal sections. R = right; L = left.

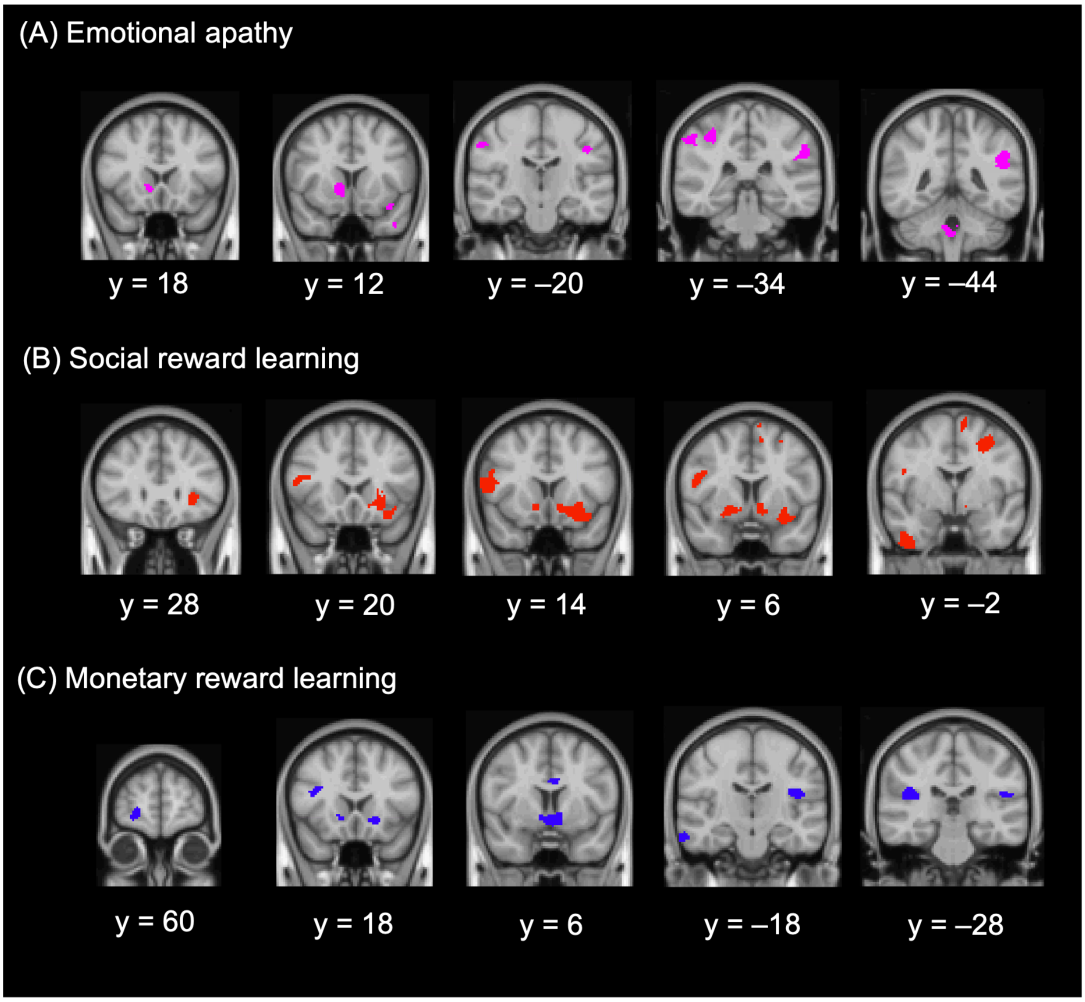

***Supplementary Table 3.*** Voxel-based morphometry results showing regions of significant grey matter intensity decrease that covary with (a) emotional apathy, (b) social reward learning and (c) monetary reward learning across all patients combined. (See also Supplementary Figure 3).

| | | Peak MNI coordinates | | | |
|---|---|---|---|---|---|
| Regions | Hemisphere | X | Y | Z | Cluster size |
| ***(a) Emotional apathy*** | | | | | |
| Supramarginal gyrus (anterior and posterior), parietal operculum cortex | L | -46 | -44 | 24 | 578 |
| Supramarginal gyrus (anterior and posterior) | R | 54 | -34 | 44 | 315 |
| Postcentral gyrus | R | 36 | -32 | 48 | 147 |
| Cerebellum | R | 8 | -48 | -42 | 143 |
| Insular cortex, orbitofrontal cortex | L | -36 | 10 | -18 | 136 |
| Nucleus accumbens, caudate, putamen | R | 12 | 10 | -2 | 134 |
| Lateral occipital cortex (superior) | L | -16 | -88 | 44 | 105 |
| ***(b) Social reward learning*** | | | | | |
| Orbitofrontal cortex, putamen, caudate, nucleus accumbens, insular | L | -22 | 14 | -14 | 827 |
| Inferior frontal gyrus, precentral gyrus | R | 58 | 10 | 4 | 407 |
| Inferior temporal gyrus (anterior) | R | 48 | -4 | -46 | 195 |
| Superior frontal gyrus, middle frontal gyrus | L | -22 | -2 | 44 | 176 |
| Insular cortex, orbitofrontal cortex, nucleus accumbens, putamen | R | 34 | 10 | -18 | 164 |
| Superior frontal gyrus, supplementary motor cortex | L | -8 | 2 | 72 | 118 |
| ***(c) Monetary reward learning*** | | | | | |
| Nucleus accumbens, subcallosal cortex | B | 0 | 2 | -16 | 569 |
| Central opercular cortex, parietal operculum cortex | L | -40 | -18 | 20 | 230 |
| Parietal operculum cortex, Heschl's gyrus, insular cortex | R | 38 | -26 | 20 | 150 |
| Anterior cingulate cortex | B | 6 | -12 | 24 | 138 |
| Frontal pole | R | 22 | 62 | -6 | 127 |
| Inferior frontal gyrus, middle frontal gyrus | R | 42 | 20 | 16 | 117 |
| Middle temporal gyrus (posterior) | R | 68 | -16 | -26 | 100 |

*Clusters were extracted voxel-wise corrected for false discovery rate at p<.05. All clusters reported t >2.63, with a cluster extent threshold of >100 contiguous voxels. L = left; R = right; B = bilateral; MNI = Montreal Neurological Institute*
